# Computational recognition of regulator genes and signature for ferroptosis with implications on immunological properties and clinical management of atopic dermatitis

**DOI:** 10.3389/fimmu.2024.1412382

**Published:** 2024-09-06

**Authors:** Lei Xu, Wenjuan Guo, Huirong Hao, Jinping Yuan, Bingxue Bai

**Affiliations:** ^1^ Department of Dermatology, The Second Affiliated Hospital of Harbin Medical University, Harbin, China; ^2^ The Key Laboratory of Myocardial Ischemia, Chinese Ministry of Education, State Key Laboratory of Frigid Zone Cardiovascular Diseases (SKLFZCD), Harbin, China

**Keywords:** ferroptosis, atopic dermatitis, immunoregulation, drug therapy, tissue-specific genes

## Abstract

**Background:**

Atopic dermatitis (AD) is a common chronic dermatitis of autoimmune origin that considerably affects the quality of life of patients. Ferroptosis, a newly regulated form of cell death, is essential for inflammation-related damage-associated molecular patterns (DAMPs). In this study, we aimed to identify ferroptosis regulators relevant to AD pathogenesis and reveal the mechanisms by which ferroptosis regulates the pathogenesis of AD.

**Methods:**

We analyzed the GEO AD cohorts (GSE16161, GSE32924, GSE107361, and GSE120721), identifying AD-related differentially expressed genes (DEGs) using edgeR. Co-expression and STRING database analyses were used to elucidate the interactions between DEGs and ferroptosis markers. Through functional enrichment analysis, we defined potential biological functions within the protein-protein interaction (PPI) network and developed FerrSig using LASSO regression. The utility of FerrSig in guiding the clinical management of AD was evaluated using the GSE32473 cohort. Subsequently, our in silico findings were confirmed, and mechanistic insights were expanded through both *in vitro* and *in vivo* studies, validating the relevance of FerrSig.

**Results:**

In the GEO AD cohort, 278 DEGs were identified, including seven ferroptosis signature genes. Co-expression analysis and STRING database review revealed a 63-node PPI network linked to cell cycle and pro-inflammatory pathways. Four ferroptosis genes (*ALOXE3*, *FABP4*, *MAP3K14*, and *EGR1*) were selected to create FerrSig, which was significantly downregulated in samples collected from patients with AD. In addition, immune-related signaling pathways were significantly differentially enriched between the stratifications of samples collected from patients with AD with high and low ferritin levels, whereas in the GSE32473 cohort, FerrSig was significantly increased in cohorts effectively treated with pimecrolimus or betamethasone. Finally, *in vitro* and *in vivo* models showed a notable FerrSig decrease in patients with AD versus healthy control. Treatment with betamethasone and tacrolimus restored FerrSig, and the magnitude of the increase in FerrSig was higher in samples collected from patients with AD with better efficacy assessments. In addition, FerrSig was significantly positively correlated with the ferroptosis inhibitors GPX4 and SLC7A11 and negatively correlated with reactive oxygen species (ROS) levels and p-STAT3/STAT3. This implies that the FerrSig signature genes may regulate ferroptosis through the JAK/STAT3 signaling pathway.

**Conclusion:**

Our study further explored the pathogenesis of AD, and FerrSig could serve as a potential biomarker for identifying AD morbidity risks and determining treatment efficacy.

## Graphical Abstract

The overview of the study design

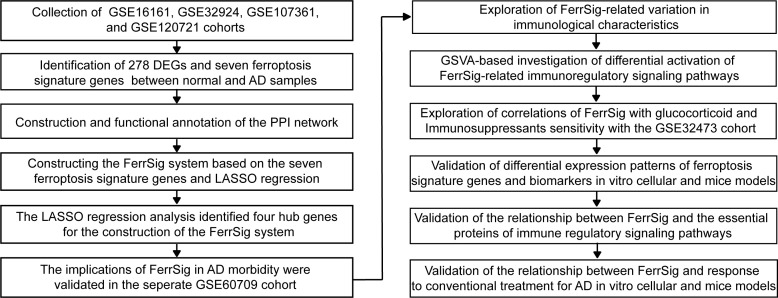

## 1 Introduction

Atopic dermatitis (AD) is the most common inflammatory skin disease. Its main clinical manifestations include lesions, rashes, and crusts on the extremities, head, and face, accompanied by intense itching and discomfort (1, 2). Some patients experience intractable itching, which seriously affects their quality of life (3). Epidemiological reports indicate that the combined prevalence of AD in the community is approximately 11%–13% (4, 5). Its prevalence is higher in children than in adults, with a combined prevalence of approximately 24% in children aged 0–5 (6). Therefore, the high prevalence of AD imposes a severe burden on public healthcare systems. In addition, several challenges remain in the clinical management of AD, including the absence of objective diagnostic tests, unavailability of publicly recognized specific biomarkers, and vulnerability to relapse (5, 7). Therefore, a more comprehensive understanding of its pathogenesis can help overcome these challenges and improve the early and accurate diagnosis and treatment of patients with AD.

Ferroptosis was first detected by Dixon et al. and is a form of cell death induced by ferrous ions and cell membrane lipid peroxidation (8). Recently, ferroptosis has been suggested to be involved in the pathophysiology of several immune-mediated diseases (9, 10). Evidence suggests that ferroptosis and the inflammatory response are mutually reinforcing. Ferroptosis triggers the intrinsic immune system by releasing inflammation-related damage-associated molecules, and immune cells stimulate an inflammatory response by recognizing the mechanisms of different patterns of cell death (11). For example, neutrophils can release extracellular traps through the ferroptosis pathway, activate toll-like receptors (TLR), and upregulate ROS levels (12). Excessive production of ROS promotes the release of pro-inflammatory cytokines and polarization of cytotoxic T cells, leading to AD morbidity and progression (13). Additionally, in psoriasis, which has a pathogenesis similar to that of AD, ROS-dependent ferroptosis is closely associated with the accumulation of inflammatory cytokines. Intervention strategies targeting ferroptosis can substantially reduce the intensity of inflammation and inhibit pathophysiological evolution (14, 15). Therefore, we speculate that ferroptosis may play an essential role in the pathophysiological development of AD. However, the mechanisms underlying ferroptosis regulation in AD have yet to be explored, and a notable knowledge gap still exists in this field. Therefore, this study attempted to reveal the critical regulators of ferroptosis in AD and their potential regulatory mechanisms, and to construct AD morbidity models for guiding its clinical management.

In this study, we identified 278 differentially expressed genes (DEGs) and seven ferroptosis signature genes in multiple GEO cohorts. Furthermore, we constructed protein-protein interaction (PPI) networks and performed functional enrichment analysis. Ferroptosis signature genes and associated DEGs were highly enriched in signaling pathways involved in immune regulation, cell cycle checkpoints, and extracellular matrix reorganization. In addition, the constructed FerrSig model could accurately identify the risk of developing AD and was suggestive of a response to glucocorticoid and immunosuppressant therapies. Finally, in both *in vitro* cellular and mouse models, we observed elevated levels of ferroptosis in AD and validated the correlation between FerrSig expression and AD morbidity and treatment response. Overall, our study revealed that ferroptosis is involved in the pathophysiological evolution of AD. In addition, FerrSig may serve as a novel biomarker with clinical applicability, and its corresponding hub genes may be potential targets for clinical interventions in AD.

## 2 Materials and methods

### 2.1 Collection and pre-processing of data

Six AD-related cohorts were obtained from the GEO database (https://www.ncbi.nlm.nih.gov/geo/). Only samples of skin tissue origin were retained in all GEO cohorts. After excluding samples from the same patient source, samples from 64 patients with AD and 34 healthy controls were included in the GSE16161, GSE32924, GSE107361, and GSE120721 cohorts. They were used to identify the DEGs and construct an AD morbidity model. The GSE60709 cohort was used as an independent external dataset to validate the accuracy and stability of the AD morbidity model. The GSE32473 cohort contained information on patients with AD treated with pimecrolimus or betamethasone. This cohort was used for the analysis of therapeutic benefits. A list of ferroptosis regulators was obtained from the “FerrDb V2” database (http://www.zhounan.org/ferrdb/) (16). The “normalizeBetweenArrays” function in the “limma” package was used to normalize expression profiles from different GEO cohorts to avoid biased results due to variations in sequencing background.

### 2.2 Identification of DEGs

To ensure accuracy, we used the Robust Rank Aggregation method to identify DEGs in samples from healthy controls and patients with AD (17). First, based on the “edgeR” algorithm, we separately evaluated the differences in expression profiles between samples from healthy controls and AD samples in GSE16161, GSE32924, GSE107361, and GSE120721 cohorts. The list of candidate DEGs was determined according to the thresholds FDR<0.05 and |logFC|>1. Furthermore, based on the “RobustRankAggreg” package, we comprehensively analyzed the LogFC and FDR values distribution of the candidate DEGs and constructed a comprehensive ranking list. Only DEGs with consistent expression patterns in at least three cohorts were selected and considered differentially expressed between samples from healthy controls and patients with AD.

### 2.3 Co-expression analysis and construction of the PPI network

First, the co-expression relationships between ferroptosis signature genes and DEGs were identified using the Spearman correlation analysis. All co-expression relationships were retained at p<0.05. Furthermore, in the STRING database, we searched for all nodes in the co-expression network (https://cn.string-db.org/) (18). With Cor>0.4 as the threshold, 63 PPI were retained. Reconstruction of PPI networks was performed using the Cytoscape software (Version 3.9.2).

### 2.4 Functional enrichment analysis

The “clusterProfiler” package was served for Gene Ontology (GO) and the Kyoto Protocol Encyclopedia of Genes and Genomes (KEGG) functional enrichment analysis (19). Signaling pathways and biological functions that met the qvalue < 0.05 were considered significant. The “org.Hs.eg.db” package was used to translate gene symbols into ensemble IDs, which enabled the gene list to be read by the “enrichGO” or “enrichKEGG” algorithm.

In addition, the gene set enrichment analysis (GSEA) can identify variations in the enrichment levels of specific signaling pathways in different sample stratifications. This strategy was applied to identify the differences in the activity of immune regulatory signaling pathways between the high- and low-FerrSig subgroups. Gene sets for characterizing the immune regulatory signaling pathways were obtained from the GSEA database (https://www.gsea-msigdb.org/gsea/).

### 2.5 Identification of the AD morbidity model with LASSO regression

Least absolute shrinkage and selection operator (LASSO) regression were performed to identify the FerrSig AD morbidity model. Firstly, with the “createDataPartition” function in the “caret” package, we randomly and equally divided the integrated GEO cohort (including GSE16161, GSE32924, GSE107361, and GSE120721) into the train and test sets. The LASSO regression was implemented based on the “glmnet” package. After 1000 iterations and validated by the 10-fold cross-validation, the optimal penalty coefficient was log(λ) = −4.35. Four ferroptosis signature genes (*ALOXE3*, *FABP4*, *MAP3K14*, and *EGR1*) had non-zero weight coefficient (Coef) values under these conditions. These are the principal components of ferroptosis signature genes in AD and are recognized as hub genes for ferroptosis in AD. FerrSig was constructed using the following formula:


$$FerrSig=\sum_{i=1}^{n}Coef(Hub\;Genes_{i})\;*\;Expression(Hub\;Genes_{i})$$

### 2.6 Immune cell infiltration and ssGSEA

ssGSEA and its derivative algorithms were used to evaluate the enrichment levels of the immune signatures for each sample. Of these, 24 gene sets were obtained from the study by Bindea et al. (20), 17 from the ImmPort database (21), and 29 from the R&D system (RndSys, https://www.rndsystems.com/). The signatures of these gene sets were evaluated by the ssGSEA method based on the “GSEA” package. In addition, 22 immune cell signatures were calculated using the CIBERSORT algorithm, a derivative of ssGSEA, and used to evaluate immune cell infiltration patterns in AD (22).

### 2.7 Statistical methods and software

The Mann-Whitney U test was used to compare the differential distribution of relevant variables between the two subtypes or subgroups. Otherwise, we used the Kruskal–Wallis test for variance analysis. Correlations between the variables were verified using Spearman’s correlation analysis. The accuracy of the morbidity model was determined with Receiver Operating Characteristic (ROC) curves and the areas under the curves (AUC).

This study was conducted using R version 4.1.1. The “pheatmap” package was used for plotting heatmaps. “ggplot2,” “ggpubr,” “ggExtra,” “plyr,” and “reshape2” packages could be used for plotting multiple figures, such as box plots and scatter diagrams. The Venn diagram was developed with the “Venn” package. The ROC curves were plotted by the “pROC” package. In addition, Perl scripts were used to preprocess the data (Strawberry-Perl-5.32.1.1).

### 2.8 Molecular biology experimental validation

#### 2.8.1 *In vitro* cell assay

Human immortalized skin keratinocytes (HaCaT cells) were purchased from Kunming Cell Bank of Type Culture Collection, Chinese Academy of Science (Kunming, China) and cultured in Dulbecco’s modified Eagle’s medium (DMEM, Gibco) supplemented with 10% fetal bovine serum (FBS; Procell) and 1% penicillin-streptomycin (v/v, Gibco) at 37°C under a humidified atmosphere of 5% CO_2_. The medium was changed every 2–3 d. HaCaT cells were cultured with 10 ng/mL of interferon-γ (IFN-γ; 10 ng/mL) and tumor necrosis factor-α (TNF-α; 10 ng/mL) for 24 h to induce the *in-vitro* AD model.

#### 2.8.2 ROS detection

HaCaT cells were seeded at a density of 2 × 10^4^ cells per well in 24-well plates. After 24 h of stimulation with 10 ng/mL of TNF-α/IFN-γ, intracellular ROS levels were assessed using a ROS detection kit (S0033S, Beyotime Biotechnology, China). Subsequently, the cells were incubated with 10 μM 2’,7’-dichlorodihydrofluorescein diacetate (DCFH-DA) in the dark at 37°C for 20 min. Subsequently, the cells were washed thrice with serum-free medium. Fluorescence was captured using a fluorescence microscope (Leica, Wetzlar, Germany).

#### 2.8.3 Animals

Female BALB/c mice (6 weeks of age, body weight: 18–22 g) provided by the Animal Laboratory Center of the Second Affiliated Hospital of Harbin Medical University were used to construct an *in vivo* AD model. They were maintained under standard conditions (temperature 21 ± 2°C; 12-h light/dark cycle) and an unlimited supply of a standard extruded pellet diet and water was provided. They were allowed a minimum of 1 week to acclimate to the colony room upon arrival. This study was conducted in accordance with the guidelines of the Declaration of Helsinki. All procedures complied with the National Institutes of Health Guide for the Care and Use of Laboratory Animals. All relevant experimental protocols were approved by the Institutional Animal Care and Use Committee of Harbin Medical University (ethical approval number: YJSDW2022-122).

Mice were divided into four groups: Control (ethanol), MC903 group (calcipotriol, Sigma-Aldrich, St. Louis, MO, USA), MC903+ tacrolimus group (0.1% TAC ointment, Protopic^®^ from Astellas Pharma Inc. Tokyo, Japan), and the MC903+ glucocorticoid group (GLU ointment: 0.05% clobetasol propionate cream, Tianyao Ltd., Alocal Pharmacy Store, Tianjin, China). Two nmol MC903 was applied topically to each ear of the mice once daily for 7 d to induce AD-like skin lesions. After fully inducing dermatitis, 1 nmol MC903 was administered daily for 7 d to sustain skin inflammation. The treatment group received topical 0.1% TAC ointment and 0.05% GLU once daily for seven consecutive days. Ear thickness and scratching frequency were measured at the indicated time (Days 0,3.5.7,10, and 15). After the indicated time (Days 0,3.5.7,10, and 15) treatment, the mice were euthanized for the following experiments. The portion of the intervention area of the auricular skin of mice was fixed with 4% paraformaldehyde for histopathological Analysis, and other remaining tissues were stored at −80 °C for mRNA and protein extraction.

#### 2.8.4 Histopathological analysis

After euthanizing and decapitating the mice, the whole ear tissues were fixed in 4% paraformaldehyde. The tissues were then dehydrated using increasing concentrations of alcohol and embedded in paraffin blocks. Finally, 5 μm sections were stained with hematoxylin and eosin (H&E) for evaluation of the epidermal thickness and inflammation. Images were taken using a light microscope (CX21; Olympus, Tokyo, Japan) to assess the histopathological changes in the mouse ears.

#### 2.8.5 RT-PCR

Tissues with a diameter of 5 mm and HaCaT cells were lysed in 1 ml TRIzol reagent (Invitrogen) to extract total RNA. An all-in-one First Strand cDNA Synthesis Kit (SM131; Sevenbio, Beijing, China) was used to reverse-transcribe RNA into cDNA. The thermal cycling apparatus was obtained from Thermo Fisher Scientific. RNA expression was determined using a real-time PCR detection system (CFX96, Bio-Rad) with SYBR Green Master Mix (SM143,Sevenbio, Beijing, China). The thermal cycling procedure was: 95°C (30s), 95°C (10s), 60°C (20s), and 72°C (25s). Results were standardized using GAPDH. And the mRNA expression levels were determined using the 2^−ΔΔCT^ method. Primers were provided by RuiBiotech (Beijing, China), and the corresponding sequences are displayed in **Table 1**.

**Table 1:** Primer sequences for RT-PCR.

Genes	Forward	Revers
hMAP3K14	GAGGAAAGAGCCCATCCACC	TCAGACCTCCCACTTGCTGT
mMAP3K14	GGGGTCCTGCTTACTGAGAAAC	TTCATTCTGTGGACCTCGCC
hFABP4	AACCTTAGATGGGGGTGTCCT	ACGCATTCCACCACCAGTTT
mFABP4	CGACAGGAAGGTGAAGAGCAT	AACACATTCCACCACCAGCTT
hALOXE3	TGTTTGCCGGCGCTGTATT	TGTTTGCTTGCCTCTGACACA
mALOXE3	CTGGTTCCTACCTGAAGGCTG	AGCGCAACAGCAAGATCTCA
hEGR1	GTTACCCCAGCCAAACCACT	GTGGGTTGGTCATGCTCACT
mEGR1	AGCCTTCGCTCACTCCACTA	AGCTGGGATTGGTAGGTGGT
hGAPDH	CTGGGCTACACTGAGCACC	AAGTGGTCGTTGAGGGCAATG
mGAPDH	GGTTGTCTCCTGCGACTTCA	TGGTCCAGGGTTCTTACTCC

#### 2.8.6 Western blot

Protein samples were extracted from the isolated mouse tissues with RIPA lysis buffer (P0013, Beyotime Biotechnology). Twenty-five micrograms of total protein of different molecular weights were separated on a 12.5% SDS-PAGE gel, which was maintained at 80 V for 30 min and 120 V for 90 min, and then transferred to PVDF membranes (3010040001; Roche Applied Science, Mannheim, Germany). The blocking of membranes were performed with TBST-5% BSA for 1 h at room temperature, and then incubated with primary antibodies GPX4 (ab125066, abcam, UK; 1:10000), XCT (ab175186, abcam, UK; 1:5000), Phospho-STAT3(Tyr705) (#9145, Cell Signaling Technology, USA; 1:2000), STAT3(10253-2-AP, Proteintech, Wuhan, Hubei, China; 1:2000), β-actin (TA-09, Zhongshanjinqiao, Inc., Beijing, China; 1:2000) overnight at 4°C. After incubation for 1 h at room temperature with secondary antibodies, ECL solution (Affinity, China) was used for image acquisition on a luminescent imaging workstation (Model 6600; Tanon, Shanghai, China). In addition, ImageJ (Version: 1.54 g, USA) was used to measure the epidermal thickness and calculate the gray values of the Western Blot strips.

#### 2.8.7 Statistical and software

All data are presented as the mean ± standard deviation (SD) and were performed with the GraphPad Prism 5.0 software (GraphPad Software, CA, USA). One-way ANOVA followed by Turkey’s multiple comparisons test and Student’s *t*-test were used to measure the differences between multiple groups and two groups. Each dependent experiment was repeated at least three times, and a *p*-value < 0.05 was considered statistically significant.

## 3 Results

### 3.1 Identification of ferroptosis signature genes in AD

**Figure 1:**
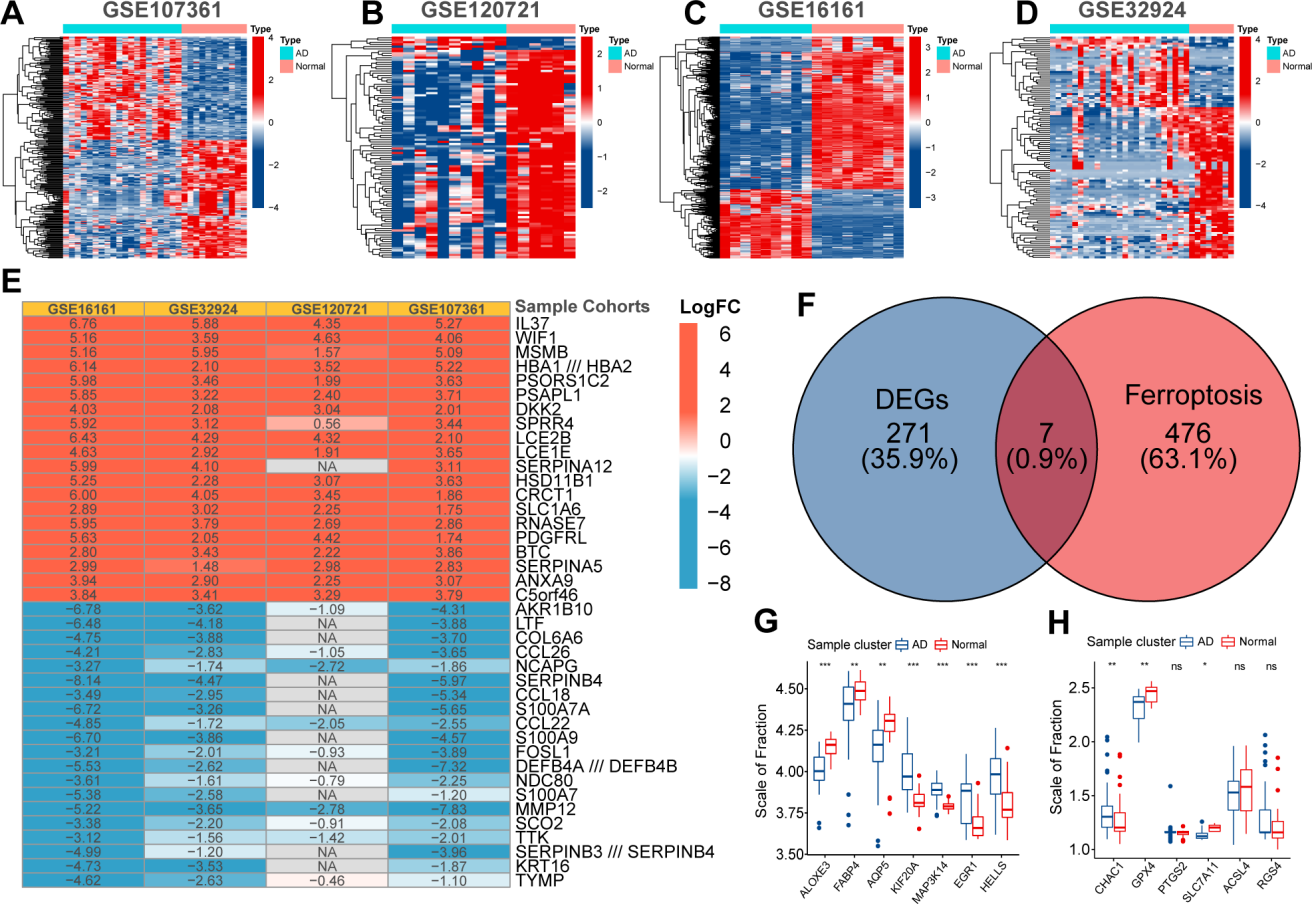
The Heatmap displayed the distribution of differentially expressed genes (DEGs) between normal and AD samples in GSE107361 **(A)**, GSE120721 **(B)**, GSE16161 **(C)**, and GSE32924 **(D)**. The robust rank aggregate heatmap presented the top 15 up-regulated and down-regulated DEGs in the ranking list **(E)**. Venn diagram presented the intersection between DEGs and ferroptosis regulators. These seven intersected genes were recognized as ferroptosis signature genes in AD **(F)**. Differential expression of ferroptosis signature genes **(G)** and ferroptosis biomarkers **(H)** between normal and AD samples. p<0.05 was indicated by "*”, p<0.01 was indicated by "**", p<0.001 was indicated by "***”.

First, the expression profiles of the GSE16161, GSE32924, GSE107361, and GSE120721 cohorts were normalized to ensure that the background expression values corresponding to each sample were consistent (**Supplementary Figure 1**). Using the thresholds of |logFC|>1 and FDR<0.05, we separately identified 3470, 1100, 1758, and 2180 DEGs between normal and AD samples in the GSE16161, GSE32924, GSE107361, and GSE120721 cohorts, respectively (**Figures 1A–D**). Next, using the Robust Rank Aggregation method, we created a comprehensive ranking list of DEGs. **Figure 1E** presented the top 15 upregulated and down-regulated DEGs. A total of 278 DEGs with consistent expression patterns in at least three cohorts were considered differentially expressed between the samples from healthy controls and patients with AD. Finally, 7/483 ferroptosis regulators simultaneously belonged to these 278 DEGs (**Figure 1F**). These are considered to be ferroptosis signature genes in AD. ALOXE3, FABP4, and AQP5 were highly expressed in samples from healthy controls, whereas KIF20A, MAP3K14, EGR1, and HELLS were highly expressed in samples from patients with AD (**Figure 1G**). Evidence suggests that ALOXE3 can increase cellular resistance to ferroptosis and FABP4 can protect cells from oxidative stress (23, 24). Similarly, EGR1 is related to GPX4 axis activity and promotes ferroptosis (25). In addition, CHAC1 and PTGS2 were significantly highly expressed in samples collected from patients with AD, whereas the expression of GPX4 and SLC7A11 was relatively low (**Figure 1H**). PTGS2 regulates the biosynthesis of COX-2, which promotes inflammation and oxidative stress. CHAC1 catalyzes glutathione catabolism, and GPX4 and SLC7A11 are protective factors in cells under oxidative stress (11). Therefore, the ferroptosis process in AD might be activated. In summary, heterogeneity in the expression patterns of ferroptosis regulators exists between samples from patients with AD and healthy controls, and activation of ferroptosis is related to the pathogenesis of AD.

### 3.2 Construction of the PPI network and functional annotation

**Figure 2:**
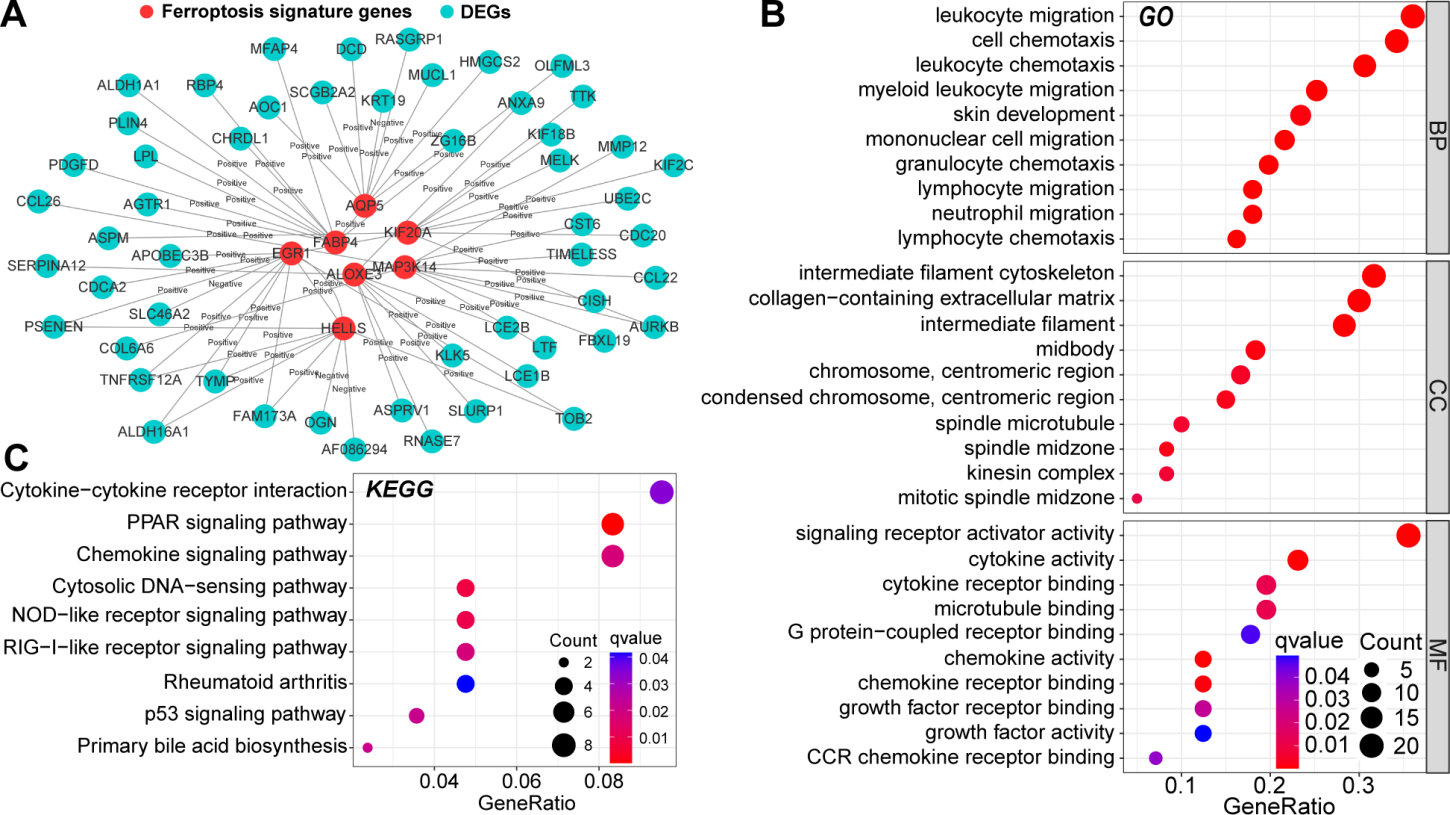
Protein-protein interaction (PPI) relationships between DEGs and ferroptosis signature genes **(A)**. Results of Gene Ontology (GO) **(B)** and the Kyoto Protocol Encyclopedia of Genes and Genomes (KEGG) **(C)** functional enrichment analysis of nodes in the PPI network. In this figure, the correlation analysis was performed with the “Spearman” correlation test. PPI with p<0.05 and Cor>0.4 in the String database could be included.

To further reveal the mechanisms by which these seven ferroptosis signature genes are involved in AD pathogenesis, we determined their co-expression relationships with the DEGs. After validation using the STRING database, 63 PPI relationships were retained (**Figure 2A**). Next, GO and KEGG functional enrichment analyses were performed on all nodes of the PPI network. These genes were highly enriched in multiple signaling pathways and molecular functions, including cell cycle regulation, immune cell chemotaxis and migration, cytokine activity, and extracellular matrix reorganization (**Figures 2B, C**). Alterations in cell-cycle regulation may be associated with ferroptosis activation (26). In addition, these ferroptosis signature genes can regulate multiple proinflammatory signaling pathways, and AD is known to be closely related to chronic inflammatory alterations. Therefore, these signature genes may regulate multiple biological pathways and have non-negligible value in AD pathogenesis.

### 3.3 Construction of the ferroptosis-related AD morbidity model

**Figure 3:**
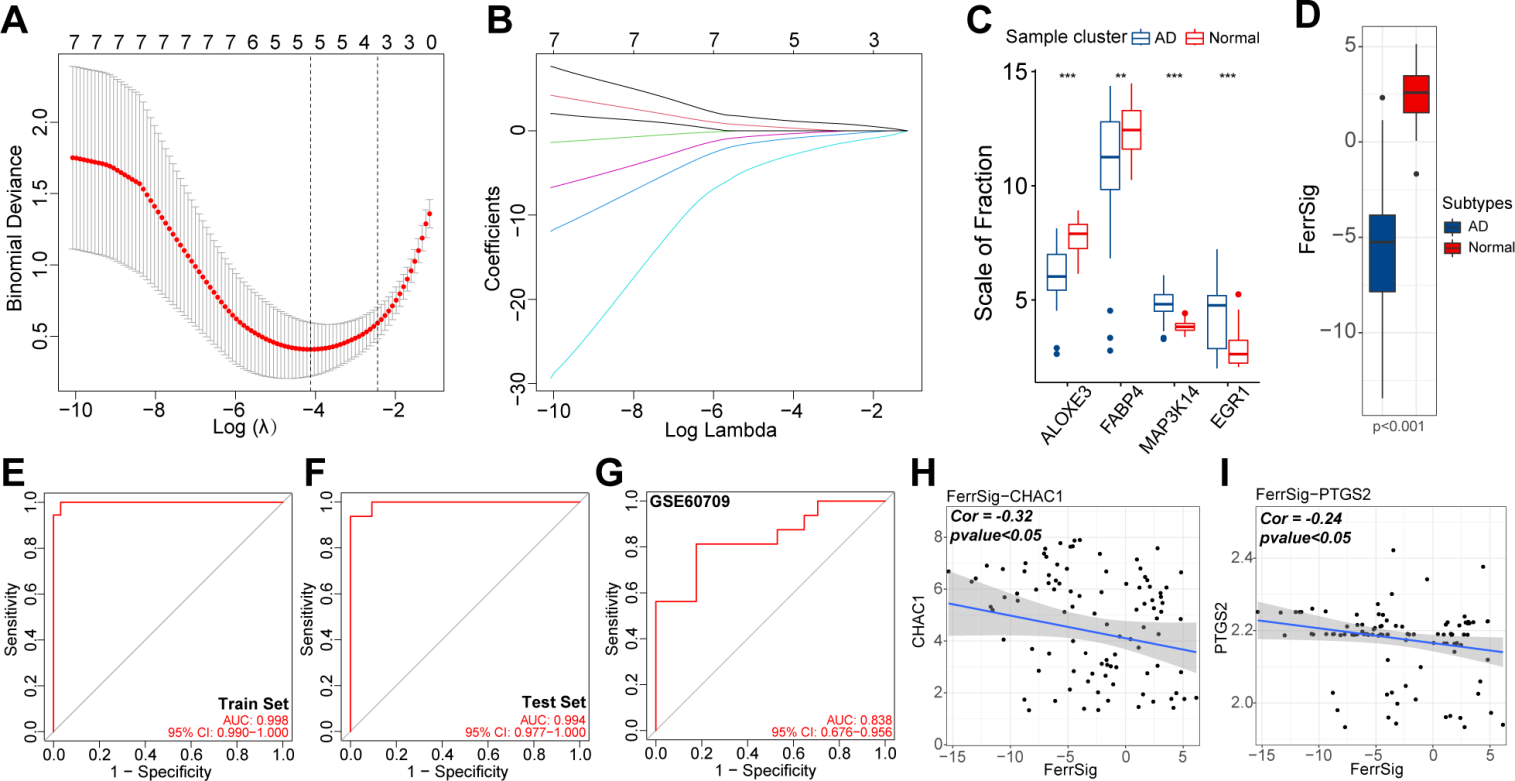
The relationship between lambda values and Binominal likelihood deviance **(A)** or variable coefficients **(B)** in the calculation of FerrSig by the least absolute shrinkage and selection operator (LASSO) regression. Differential expression of four LASSO hub genes between normal and AD samples **(C)**. Differential distribution in FerrSig between normal and AD samples **(D)**. ROC curves of FerrSig in the train set **(E)**, test set **(F)**, or the GSE60709 cohort **(G)**. Correlations between FerrSig and the expression of CHAC1 **(H)** and PTGS2 **(I)**. In this figure, the correlation analysis was performed with the “Spearman” correlation test. In the box plots, p<0.01 was indicated by "**", p<0.001 was indicated by "***”, and the statistical analysis was performed by the Mann-Whitney U test.

To quantify the risk of AD morbidity, we developed a genetic model with LASSO regression. After 10-fold cross-validation, the optimal penalty coefficient was log(λ) = −4.35 (**Figure 3A**). Under these conditions, four ferroptosis signature genes (*ALOXE3, FABP4, MAP3K14*, and *EGR1*) had nonzero weight coefficient (Coef) values (**Figure 3B**). Therefore, they were considered hub genes with the most significant effect on AD morbidity and were used in the construction of FerrSig. MAP3K14 and EGR1 were highly expressed in the AD subgroup, whereas *ALOXE3* and *FABP4* were highly expressed in samples from healthy controls (**Figure 3C**). As shown in **Supplementary Table 1**, The Coef values for *ALOXE3* and *FABP4* were positive, whereas those for *MAP3K14* and *EGR1* were negative. Therefore, FerrSig expression was downregulated in the AD subgroup (**Figure 3D**). For the ROC curves, the AUC values corresponding to FerrSig were 0.998 and 0.994 for the training and test sets, respectively (**Figures 3E, F**). Furthermore, a separate GEO cohort was used to verify the accuracy of FerrSig. In the GSE60709 cohort, FerrSig showed an AUC of 0.838 (**Figure 3G**). Therefore, the predictive capability of FerrSig was stable and its accuracy was relatively reliable across sample cohorts from different sources and origins. Furthermore, FerrSig was significantly negatively correlated with CHAC1 and PTGS2, further supporting a more activated ferroptotic process in AD (**Figures 3H, I**). In summary, FerrSig may serve as a potential quantitative marker of ferroptosis. In addition, FerrSig may be a reliable biomarker for the risk of AD morbidity.

### 3.4 The immune landscape analysis

Ferroptosis is an important mechanism in oxidative stress-related cell death. In addition, the inflammatory response was positively correlated with oxidative stress levels. In addition, the identified ferroptosis signature genes were highly enriched in multiple immune-related signaling pathways (**Figures 2B, C**). To clarify the mechanism by which ferroptosis participates in the pathogenesis of AD, we attempted to depict the immune landscape of AD and reveal the immunological significance of FerrSig.

**Figure 4:**
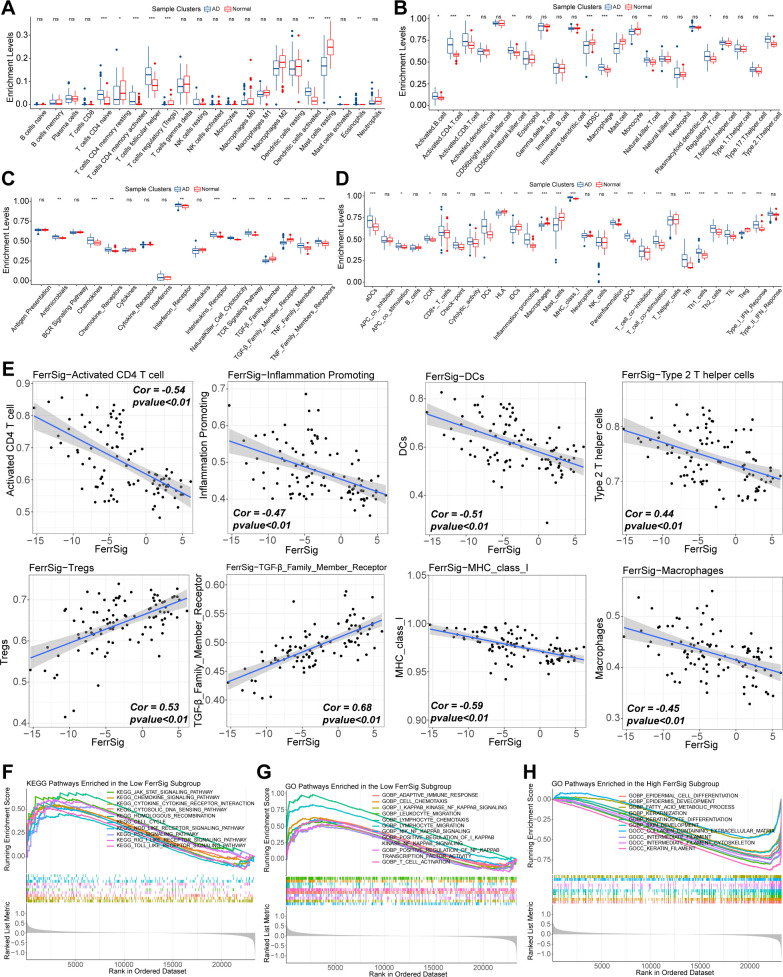
Differences in the enrichment levels of immune signatures of CIBERSORT **(A)**, works of Bindea et al. **(B)**, ImmPort database **(C)**, and the RndSys **(D)** between normal and AD samples. Correlations of FerrSig with immune signatures whose Cor was larger than 0.4 **(E)**. Top 10 highly enriched KEGG signaling pathways in the low FerrSig subgroups **(F)**. Top 10 highly enriched GO signaling pathways in the low FerrSig subgroups **(G)** and high FerrSig subgroups **(H)**. In this figure, the correlation analysis was performed with the “Spearman” correlation test. In the box plots, p<0.05 was indicated by "*”, p<0.01 was indicated by "**", p<0.001 was indicated by "***”, and the statistical analysis was performed by the Mann-Whitney U test.

First, we depicted the differences in immune cell infiltration patterns between normal and AD subgroups. As shown in **Figures 4A–D**, pro-inflammatory cells, including activated CD4/CD8 cells, Tfh cells, activated dendritic cells, natural killer cells, and B cells, were highly infiltrated in the AD subgroup. In contrast, the proportion of immunomodulatory cells, such as regulatory T cells (Tregs) and multiple resting immune cells, was significantly higher in the samples from healthy controls. In addition, the levels of various costimulatory factors, cytokines, chemokines, interleukins, and inflammation-promoting receptor signatures were significantly higher in the AD subgroup (**Figure 4C**). These results suggest that AD is characterized by a distinctive abnormal activation of inflammatory activities. Furthermore, we evaluated the correlation between FerrSig expression and the immune signatures. The enrichment levels of macrophages, DCs, activated CD4 + T cells, inflammatory activity, major histocompatibility complex class I cells, and Th2 cells were negatively correlated with FerrSig (**Figure 4E**). However, the activities of transforming growth factor-β, and Tregs were positively correlated with FerrSig (**Figure 4E**). Therefore, FerrSig may be negatively correlated with inflammatory activity. In addition, we noted that JAK/STAT, NF-κB, p53, NOD-receptor, and TLR signaling pathways were highly enriched in the low FerrSig subgroup (**Figures 4F–H**). However, in the high-ferritin subgroup, only signaling pathways related to normal cell metabolism and skin development were highly enriched. This indicated that low ferritin levels were closer to the pathological state of AD, further supporting the accuracy of our AD morbidity model. In summary, FerrSig could serve as a biomarker of the immune status in AD. This might be attributed to the possibility that ferroptosis and immune regulation share signaling pathways.

### 3.5 Therapeutic benefit of FerrSig

**Figure 5:**
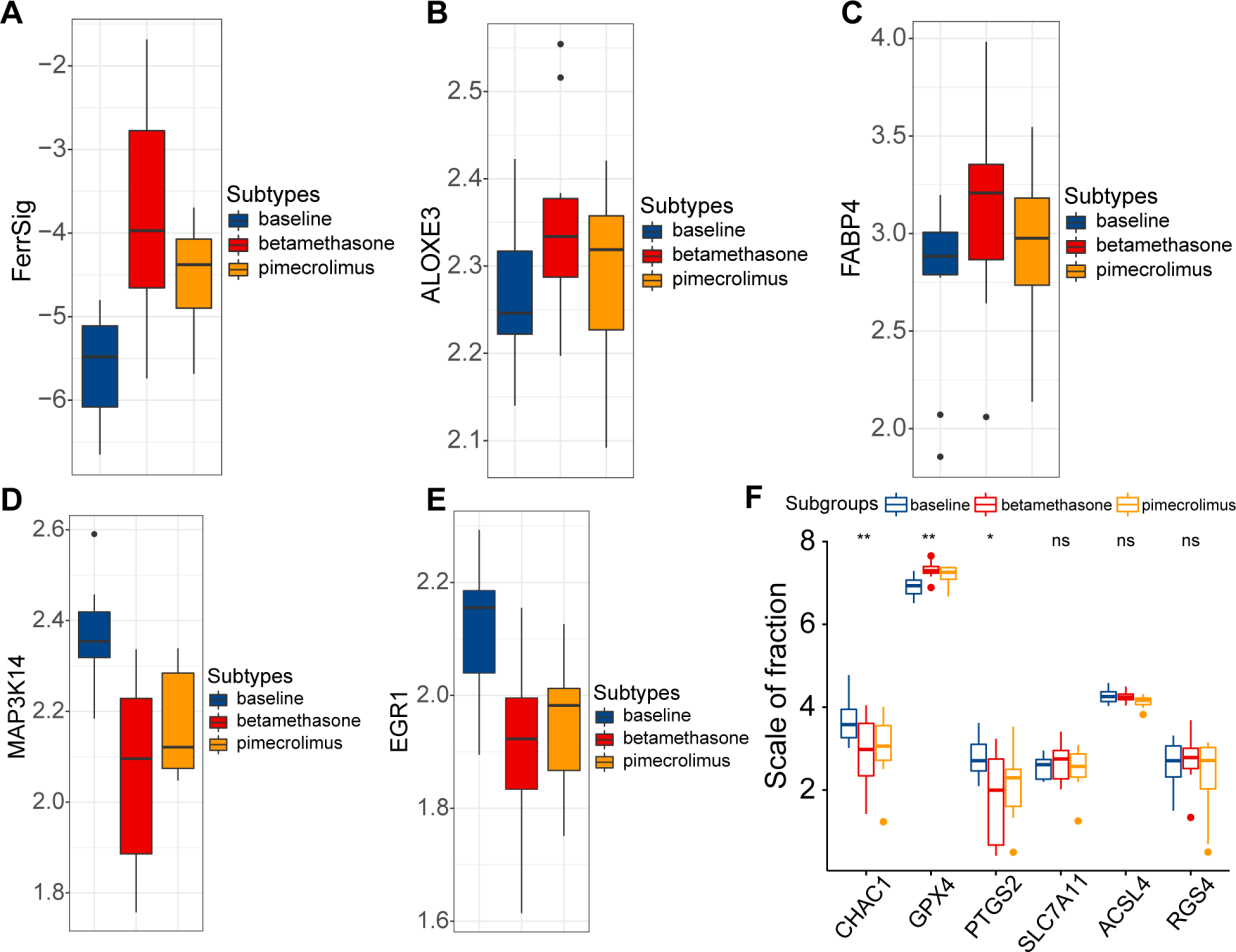
Differences in FerrSig **(A)** and expression of ALOXE3 **(B)**, FABP4 **(C)**, MAP3K14 **(D)**, EGR1 **(E)**, and ferroptosis biomarkers **(F)** between the baseline, pimecrolimus, and betamethasone subgroups. In the box plots, p<0.05 was indicated by "*”, p<0.01 was indicated by "**", and the statistical analysis was performed by the Kruskal-Willis test.

Finally, we evaluated the relationship between FerrSig and the therapeutic response in AD. Currently, the conventional therapeutic agents for AD are glucocorticoids and immunosuppressants. Therefore, we further explored this issue in the GSE32473 cohort, which received pimecrolimus or betamethasone. After 22 d of treatment, the FerrSig levels were significantly elevated under improved conditions (**Figure 5A**). In addition, we noted that the expression of ALOXE3 and FABP4 sequentially increased in the baseline, pimecrolimus, and betamethasone subgroups, whereas the expression of MAP3K14 and EGR1 sequentially decreased (**Figures 5B–E**). Compared to **Figure 3C**, the expression profile of ferroptosis signature genes in the treated subgroups tended toward samples from healthy controls compared to samples at baseline. In addition, the expression of CHAC1 and PTGS2 significantly decreased in the treated subgroups, whereas GPX4 expression was upregulated (**Figure 5F**). These results indicate that conventional AD therapy may inhibit ferroptosis. In addition, FerrSig may serve as a biomarker of treatment response, and its elevation signified that these patients responded well to clinical management.

### 3.6 Validation by *in vivo* and *in vitro* experiments

**Figure 6:**
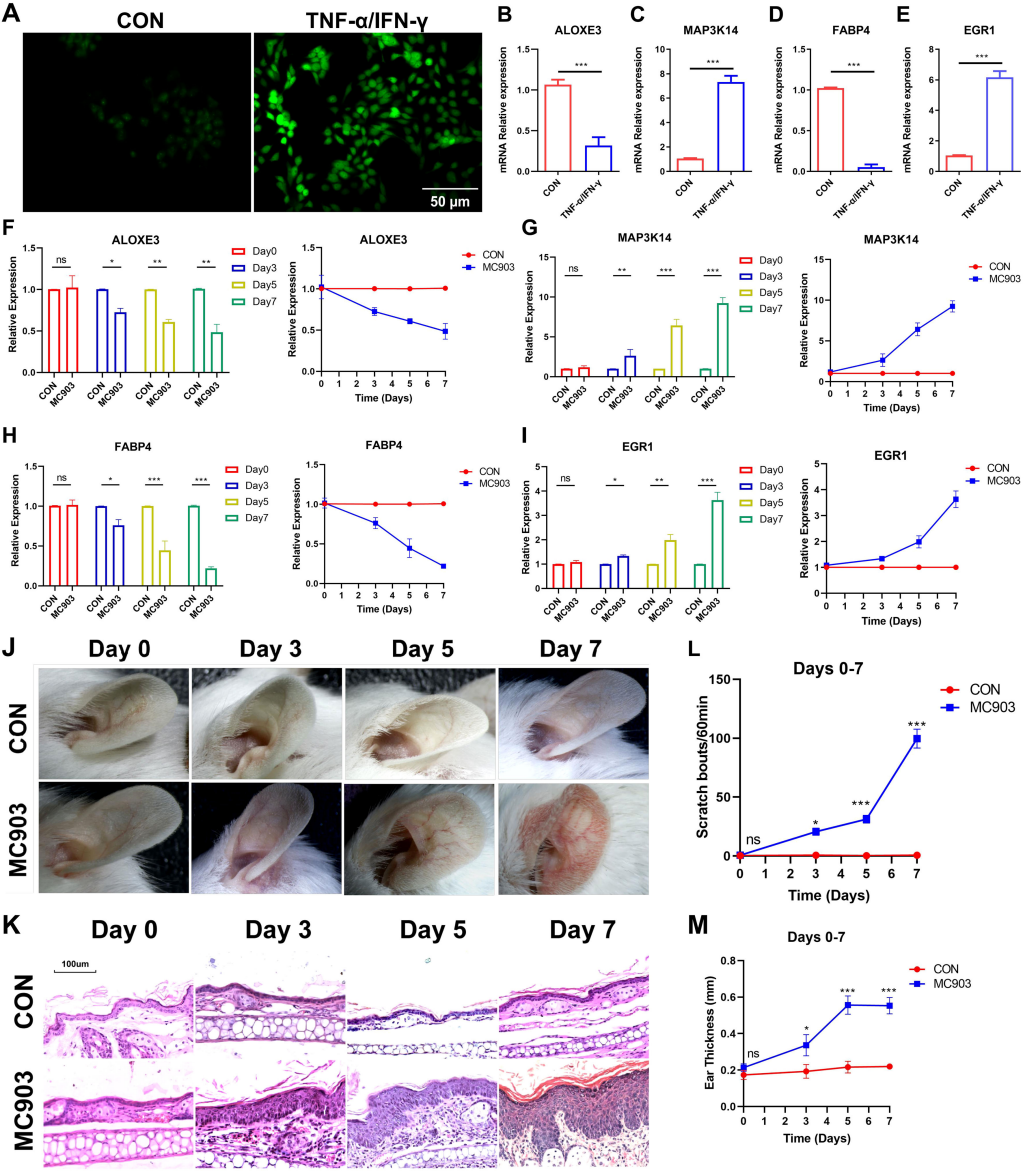
ROS fluorescent staining **(A)**. Differential expression of ALOXE3 **(B)**, MAP3K14 **(C)**, FABP4 **(D)**, and EGR1 **(E)** between CON and TNF-α/IFN-γ subgroups (MC903) in the cellular model. Differential expression of ALOXE3 **(F)**, MAP3K14 **(G)**, FABP4 **(H)**, and EGR1 **(I)** betweenthe CON and MC903 in the time points of 0 days, 3 days, 5 days, and 7 days of the mice model. Photograph of a large view of the intervention area of the mouse auricle in the time points of 0 days, 3 days, 5 days, and 7 days **(J)**. Photograph of H&E staining of mouse auricular intervention area tissue in the time points of 0 days, 3 days, 5 days, and 7 days **(K)**. Differences in scratching frequency **(L)** and auricular thickness **(M)** between CON and MC903 subgroups in the time points of 0 days, 3 days, 5 days, and 7 days. In this figure, p<0.05 was indicated by "*”, p<0.01 was indicated by "**", p<0.001 was indicated by "***”.

The FerrSig model was validated using HaCaT cells. As shown in **Figure 6A**, after 24 h of stimulation with 10 ng/mL of TNF-α/IFN-γ, the ROS level was significantly increased. We noted that MAP3K14 and EGR1 expression levels were about 8- and 7-fold higher in the TNF-α/IFN-γ subgroup, whereas the expression of ALOXE3 and FABP4 was around 1/4 and 1/10 of that of the control (**Figures 6B–E**). These results are consistent with those shown in **Figure 3C**, further supporting the reliability of the FerrSig model in predicting AD morbidity.

Second, an *in vivo* AD morbidity model was constructed using Female BALB/c mice. First, we detected changes in the expression of ferroptosis signature genes at four time points: days 0, 3, 5, and 7. ALOXE3 and FABP4 were downregulated with increasing stimulation duration, and MAP3K14 and EGR1 expression was upregulated, which was consistent with the results in the cellular model (**Figures 6F–I**). Second, with the prolongation of stimulation, the ear thickness of the mice also gradually and significantly increased, as did the frequency of scratching (**Figures 6J–M**, **Supplementary Figures S2, S3**). In addition, compared to the control, the auricular skin of AD mice presented significant edema, dryness, and erythema in the auricular skin (**Figure 6K**, **Supplementary Figure S3**). These lesion characteristics became progressively more pronounced with increasing stimulation duration. These results further validate FerrSig as a reliable biomarker for predicting the pathogenesis and severity of AD.

**Figure 7:**
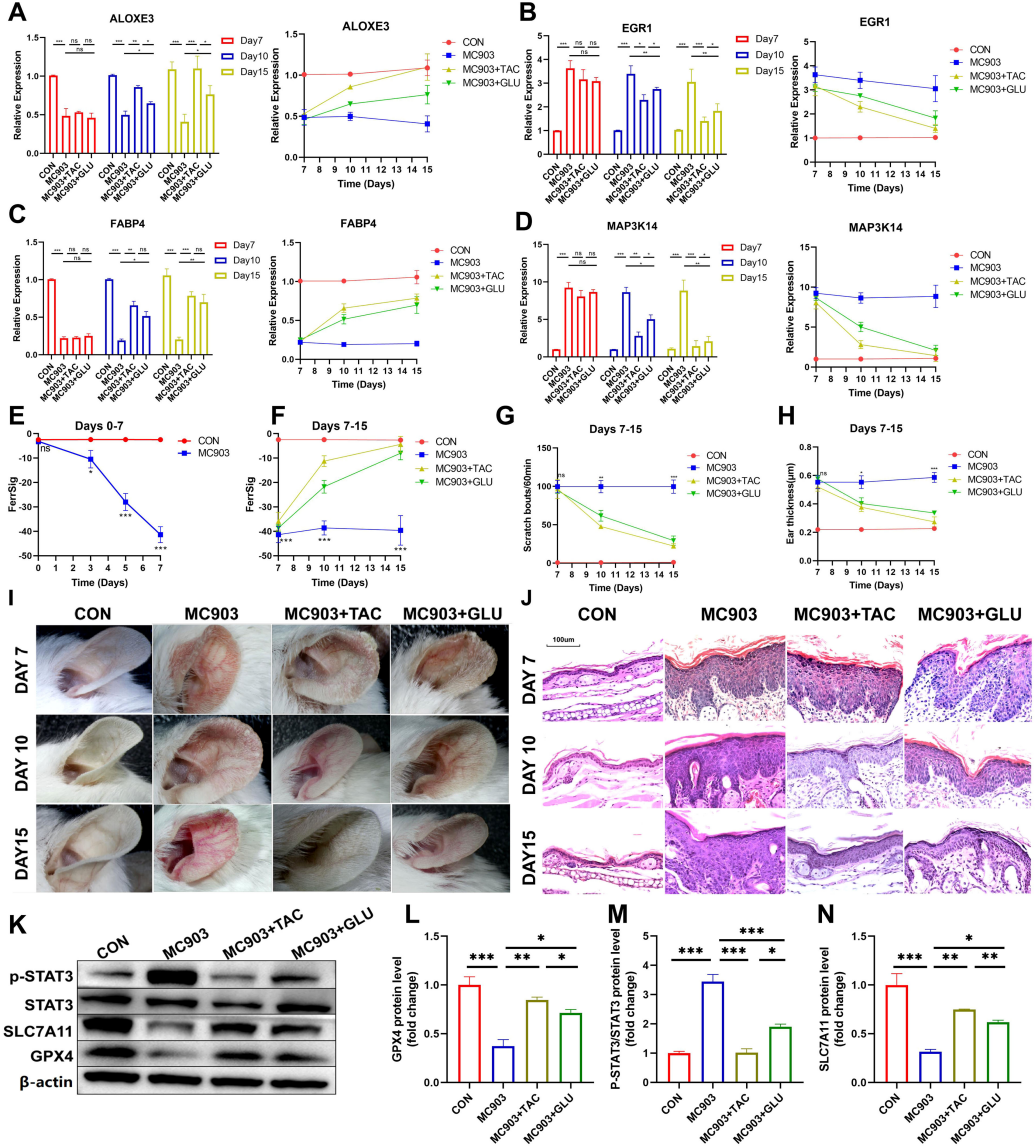
Differential expression of ALOXE3 **(A)**, EGR1 **(B)**, FABP4 **(C)**, and MAP3K14 **(D)** between the CON, MC903, MC903+TAC, and MC903+GLU subgroups between the CON, MC903, MC903+TAC, and MC903+GLU subgroups in the time points of 7 days, 10 days, and 15 days. Differences in FerrSig between the CON and MC903 subgroups in the time points of 0 days, 3 days, 5 days, and 7 days of the mice model **(E)**. Differences in FerrSig between the CON, MC903, MC903+TAC, and MC903+GLU subgroups in the time points of 7 days, 10 days, and 15 days of the mice model **(F)**. Differences in scratching frequency **(G)** and ear thickness **(H)** between the CON, MC903, MC903+TAC, and MC903+GLU subgroups in the time points of 7 days, 10 days, and 15 days. Photograph of a large view of the intervention area of the mouse auricle in the time points of 7 days, 10 days, and 15 days **(I)**. Photograph of H&E staining of mouse auricular intervention area tissue in the time points of 7 days, 10 days, and 15 days **(J)**. Differences in p-STAT3, STAT3, SLC7A11, GPX4, and β-actin protein expression between the CON, MC903, MC903+TAC, and MC903+GLU subgroups in the mice model **(K)**. Relative expression of GPX4 **(L)**, p-STAT3/STAT3 **(M)**, and SLC7A11 **(N)** between the CON, MC903, MC903+TAC, and MC903+GLU subgroups in the mice model.In this figure, p<0.05 was indicated by "*”, p<0.01 was indicated by "**", p<0.001 was indicated by "***”.

Next, when the *in vivo* AD model was successfully constructed (day 7), two groups of AD mice were treated with tacrolimus or betamethasone. We similarly examined the differences in the expression of ferroptosis signature genes, auricular thickness, and number of scratches on days 10 and 15. The results showed that the symptoms of AD in the betamethasone- and tamoxifen-treated groups improved significantly by day 10, as evidenced by a reduction in skin lesions and frequency of scratching (**Figures 7G, H**). These changes were more pronounced as treatment progressed to day 15. Notably, the tacrolimus subgroup showed better improvement in appearance than the betamethasone subgroup (**Figure 7I**, **Supplementary Figures S4A–C**). Hematoxylin and eosin (H&E) staining revealed significant epidermal thickening, parakeratotic hyperkeratosis, epidermal hyperplasia, lymphocyte cytosis, and spongiogenesis in the skin of AD mice. Similarly, compared with betamethasone, tacrolimus-treated AD mice had epidermal thickness closer to that of healthy controls and showed a more pronounced improvement in epidermal thickening, parakeratotic hyperkeratosis, epidermal hyperplasia, lymphocyte cytosis, and spongiogenesis (**Figure 7J**, **Supplementary Figures S4D–F**). Therefore, we conclude that clinical regression was better in tacrolimus-treated AD mice than in mice treated with betamethasone. Furthermore, ALOXE3 and FABP4 were upregulated over time in both the betamethasone- and tacrolimus-treated subgroups, and the expression of MAP3K14 and EGR1 exhibited a progressive decrease (**Figures 7A–D**). Notably, tacrolimus-treated AD mice had significantly higher FerSig levels than untreated AD mice, second only to normal controls. Relatively poorly regressed betamethasone-treated mice showed a lower increase in FerrSig, but it was also significantly higher than that in AD mice (**Figure 7F**). In summary, FerrSig mice responded well to AD treatment.

Finally, we validated signaling pathways potentially relevant to FerrSig. Based on **Figure 4F**, we noted that the JAK/STAT3 signaling pathway changed most significantly in the low-ferritin subgroup. We noted that p-STAT3/STAT3 was significantly elevated in AD mice, implying that STAT3 phosphorylation was significantly activated, and the activity of the JAK/STAT3 signaling pathway was markedly upregulated (**Figures 7K, M**). SLC7A11 and GPX4 were significantly downregulated in AD mice, suggesting the activation of ferroptosis (**Figures 7K, L, N**). In addition, in both betamethasone- and tacrolimus-treated AD mice, we observed a rebound in GPX4 and SLC7A11 as well as a decrease in p-STAT3/STAT3 (**Figures 7K–N**). The magnitude of change in these metrics was greater in tacrolimus-treated AD mice with higher ferritin levels and better clinical regression (**Figures 7E, F**). Therefore, the FerrSig signature gene may regulate ferroptosis by participating in the JAK/STAT3 signaling pathway and regulating STAT3 phosphorylation, which makes FerrSig clinically significant for predicting AD pathogenesis and treatment regression.

## 4 Discussion

We thoroughly analyzed 278 DEGs in the samples collected from healthy controls and patients with AD. After extensive investigations, we identified seven signature genes directly linked to ferroptosis. Functional enrichment analysis revealed that these seven genes were significantly enriched in cell cycle checkpoints and immune regulation-related signaling pathways, which are crucial for ferroptosis and the development and progression of AD. To provide accurate results regarding the risk of AD, evolution of immunological characteristics, and response to traditional AD therapy, we developed a FerrSig model. The accuracy of this model was validated using a test set and independent GEO cohort. Finally, ferritin levels were significantly decreased in the AD subgroup in both *in vitro* cell and mouse models. In addition, in mice that responded better to betamethasone and tacrolimus therapy, we observed a significant increase in FerrSig and a corresponding significant decrease in ferroptosis. In summary, our study reveals the mechanism by which ferroptosis regulates the pathophysiological development of AD. Furthermore, FerrSig has the potential to be utilized as a new biomarker with clinical relevance, and the related hub genes may have important biological implications in the pathophysiological changes of AD and could be promising targets for clinical intervention.

Ferroptosis is an emerging form of immunogenic cell death (ICD) that is characterized by iron-dependent lipid peroxidation (4, 27). Inflammation-related DAMPs should be closely associated with the activation and outbreak of inflammation in pathological states (28, 29). For example, the pattern recognition receptor (PRR) TLR4 can recognize or mediate ferroptosis-related cell death to induce myocardial tissue fibrosis and ischemia-reperfusion injury through biological signaling such as Trif or TRIM44 (30, 31). Also, ferroptosis is widespread in the pathogenesis of auto-immunogenic diseases (10, 32, 33). Of these, RA and psoriasis have similar pathogenic causative factors as AD and are essentially chronic sterile inflammatory diseases induced by abnormal oxidative stress states (2, 34–36). Notably, ROS in RA is closely associated with lipid peroxidation and abnormal mitochondrial function and can increment inflammation by inducing the generation of TNF-α, interleukin-6 (IL-6), and IL-1β (37). Similarly, the upregulation of PTGS2 and TFRC expression and decreased FTL, GPX4, and FTH1 mRNA levels were observed in psoriasis samples (15). Blocking lipid peroxidation could remarkably inhibit ferroptosis and reduce cytokine production, including TNF-α, IL-6, IL-1α, IL-1β, IL-17, IL-22, IL-23, and IL25, as well as related products like MDA and 4-HNE (15, 38).. Similarly, PRR and cytokines play essential roles in the pathogenesis of AD. For example, TLR2 can induce p38 kinase phosphorylation, which drives monocytes to express high-affinity IgE receptors and exacerbates AD symptoms (39, 40). Cytokines such as IL-25 and IL-33 can stimulate Langerhans cell activation and Th2 cell polarization, thereby mediating innate immune responses in AD pathogenesis (41, 42). Th2 cells and related pathways are important factors that induce ICD (43). Therefore, we speculated that ferroptosis might be activated in patients with AD with a background similar to that of autoimmune dysregulation. In our study, significant hyper-expression of CHAC1 and decreased expression of GPX4 and SLC7A11 were observed in samples from patients with AD. In addition, ROS levels were markedly increased in AD cells and tissues. These results support our hypotheses. In addition, the seven identified AD signature genes were highly enriched in cell cycle checkpoints and multiple immunoregulatory signaling pathways. The cell cycle checkpoint is related to the activity of the p53 signaling pathway and is one of the signals that induce ferroptosis (26). From the results of KEGG enrichment analysis, we noted that seven AD signature genes were also highly enriched in the p53 signaling pathway. In summary, ferroptosis may be closely associated with abnormal immune regulation in patients with AD. This may be involved in the pathophysiology of AD pathophysiology.

Based on the machine learning approach of LASSO regression, we identified four hub genes (*ALOXE3, FABP4, MAP3K14*, and *EGR1*) to construct the FerrSig model. ALOXE3 and FABP4 are key enzymes that transport the proteins associated with lipid metabolism. Previous studies have indicated that the pathogenesis of AD is accompanied by the inhibition of lipid metabolism (44, 45). In our study, FABP4 and ALOXE3 were also significantly hypoexpressed in samples from patients with AD. Subsequently, EGR1 and MAP3K14 are regulators of the NF-κB signaling pathway, and their high expression promotes the ferroptosis process (46). Additionally, EGR1, a critical transcription factor, has been repeatedly is highly expressed in AD tissues. EGR1 can regulate the inflammatory response in the pathogenesis of AD by modulating the IL4, MAPK, and TSLP signaling pathways (47–49). In our study, MAP3K14 and EGR1 were negatively correlated, whereas ALOXE3 and FABP4 were positively correlated with FerrSig. The overall manifestation was a significant decrease in the FerrSig levels in patients with AD. Thus, FerrSig reliably predicted the risk of AD Morbidity. Furthermore, we found that the JAK/STAT, NF-κB, NOD-receptor, and TLR signaling pathways were highly enriched in the low FerrSig subgroup. These inflammatory signaling pathways are closely associated with ferroptosis. For example, IFN-γ signaling can inhibit SLC7A11 expression via the JAK/STAT signaling pathway, which induces ferroptosis cell death (50). Our study also observed the activation of IFN-γ signaling and downregulation of SLC7A11 expression in AD. The activity of the NF-κB signaling pathway is closely related to the generation of cytokines such as IL-6 and IL-1β, as well as the infiltration levels of Th22 cells. It also influences the GPX4 axis and induce ferroptosis (51). In our study, essential proteins of these signaling pathways were highly expressed in AD tissues, suggesting that these pathways are highly activated during AD pathogenesis. Thus, these shared signaling pathways may be important mechanisms by which ferroptosis regulates the pathology of AD.

Finally, we investigated the correlation between FerrSig levels and sensitivity to conventional AD treatments. The results showed a significant increase in FerrSig levels in the treatment subgroup. These results were validated using a mouse model. In addition, the expression of CHAC1 and PTGS2 significantly decreased in the treated subgroups, whereas GPX4 expression was upregulated. Therefore, glucocorticoids and immunosuppressants may have inhibited the ferroptosis in AD. This may be related to the mechanisms underlying the effects of these two types of agents. Both cortisol and immunosuppressants can significantly suppress inflammatory responses and oxidative stress (52, 53). Furthermore, these agents have significant inhibitory effects on inflammatory signaling pathways, including JAK/STAT, NF-κB, and TLR (54–57). In the treatment subgroup, we also detected the downregulation of JAK/STAT signaling pathway activity. These molecular mechanisms may underlie the therapeutic predictive capabilities of FerrSig. In addition, upregulated FerrSig-related hub genes have the potential to serve as clinical intervention targets. MAP3K14 is the upstream kinase of NF-κB (58). EGR1 mainly acts on the GPX4 axis, and its overexpression can downregulate GPX4, thereby inducing ferroptosis (25). Strategies targeting EGR1 have been validated for selected diseases. Ai et al. postponed the progression of renal fibrosis by suppressing the expression of EGR1 (59). Whether these targets can be used as new clinical intervention paradigms for AD requires further investigation.

In this study, the FerrSig model identified ferroptosis-related genes combined with DEGs and PPI networks, reflecting genetic changes in onset of AD. FerrSig can serve as an early diagnostic biomarker by detecting gene expression to identify high-risk individuals, especially those with a family history or environmental risk factors, allowing early diagnosis or intervention to reduce incidence. The study showed that the FerrSig scores significantly increased after treatment with tacrolimus or betamethasone, indicating their potential for monitoring treatment efficacy and guiding personalized treatment plans. FerrSig scores can help evaluate patient responses to various treatments, optimize therapeutic strategies, and minimize adverse effects. Future integration with other clinical indicators, such as skin lesion scores and serum IgE levels, can provide a comprehensive assessment to predict prognosis and relapse risk, identify patients at high risk of chronicity or recurrence, and enable early intervention and management to reduce recurrence and disease burden.

This study has some limitations. First, our study was conducted on normal and AD sample cohorts and focused on the mechanisms related to ferroptosis in the pathogenesis of AD. However, due to the limited sample size available in the GEO database, we could not perform further stratification and subtype identification of the AD samples. Therefore, the biological functions of the ferroptosis marker genes identified during AD progression need to be further explored. In the future, we hope to further validate the prognostic predictive capability by collecting clinical samples, performing RNA-seq, and counting their prognostic and therapeutic information. Next, we aimed to individually interfere with the FerrSig Hub genes in both *in vitro* and *in vivo* models to further refine their mechanisms of AD pathogenesis, prognosis, and therapy. In addition, heterogeneity and complexity between samples are non-negligible factors, and relevant studies have confirmed that heterogeneity could affect reactions to treatment (60). However, our study provides new insights into the pathophysiology and treatment of AD, with important implications.

## 5 Conclusion

Our study complements the exploration of the pathophysiological mechanisms underlying AD. We explored the potential interactions between ferroptosis and AD immunomodulation by revealing the changes in the activity of signaling pathways. These findings have led to a more comprehensive and precise understanding of the pathogenesis of AD. In addition, FerrSig may be a valid biomarker for recognizing the morbidity risk of AD and its response to conventional therapies. Further clarification and refinement of the biological function of ferroptosis in AD progression are needed. Through prospective studies, we can better understand pathophysiological changes and provide clinical benefits to patients with AD.

## Data Availability

The original contributions presented in the study are included in the article/**Supplementary Material**. Further inquiries can be directed to the corresponding author.
