# Supplementary material for: Computational recognition of regulator genes and signature for ferroptosis with implications on immunological properties and clinical management of atopic dermatitis
Source: Front Immunol. 2024 Sep 6;15:1412382. doi: 10.3389/fimmu.2024.1412382 (PMC11412816; doi:10.3389/fimmu.2024.1412382)

Supplementary Figure S1 | Variations in background gene expression between samples of each GEO cohort before and after the normalization.

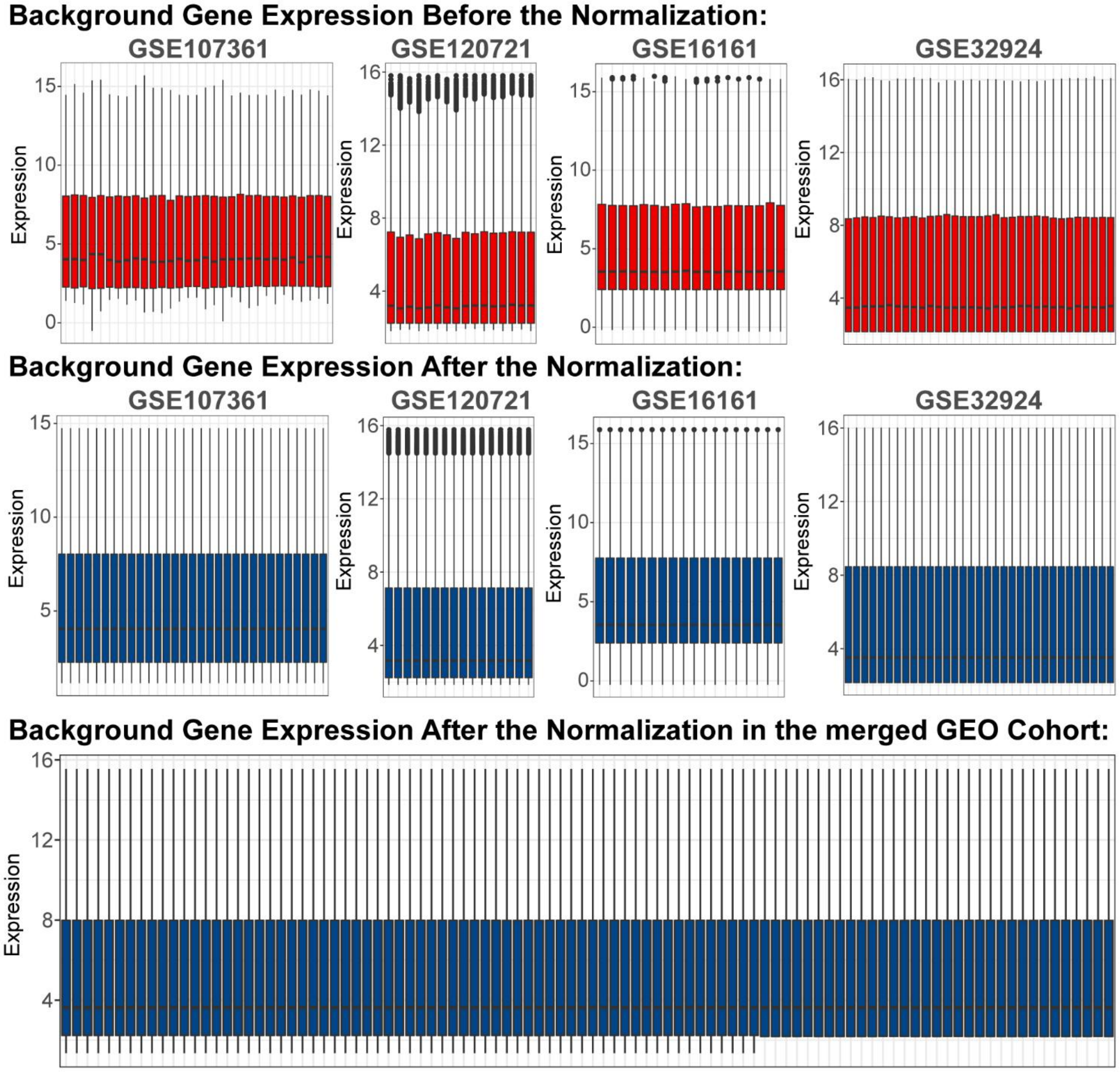

Supplementary Figure S2 | Photograph of a large view of the intervention area of the mouse auricle between CON and MC903 subgroups in the time points of 0 days, 3 days, 5 days, and 7 days. In this figure, each subgroup contains three independent replicates.

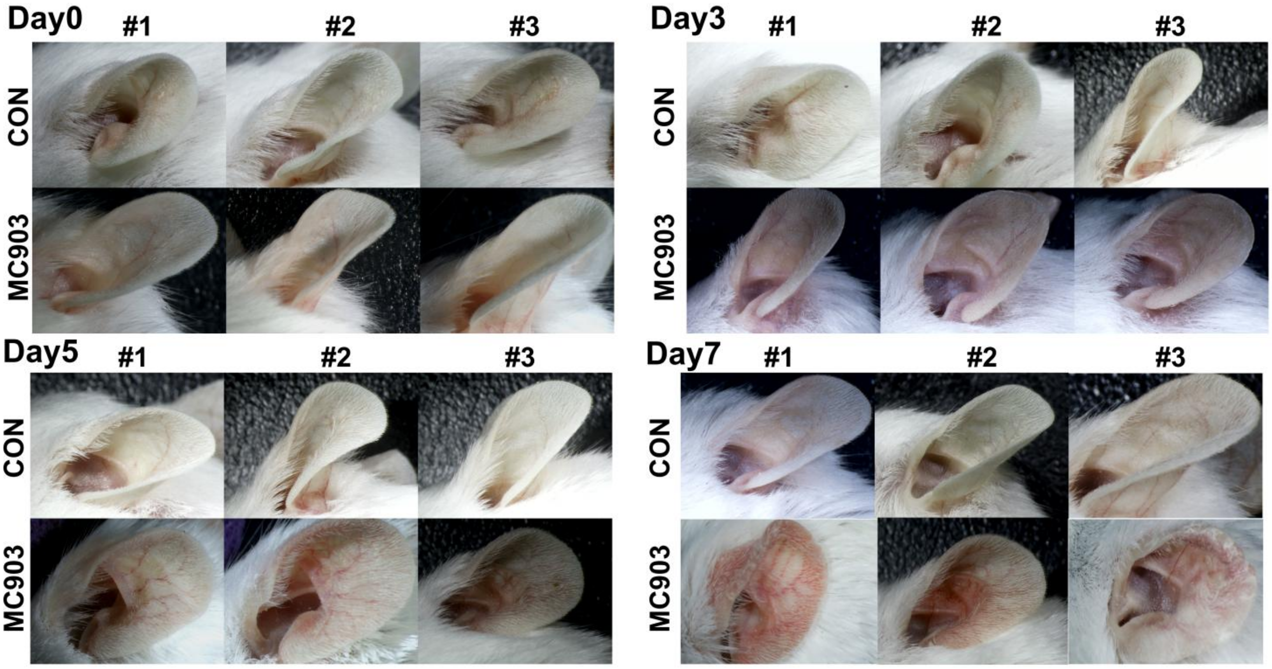

Supplementary Figure S3 | Photograph of H&E staining of mouse auricular intervention area tissue between CON and MC903 subgroups in the time points of 0 days, 3 days, 5 days, and 7 days. In this figure, each subgroup contains three independent replicates.

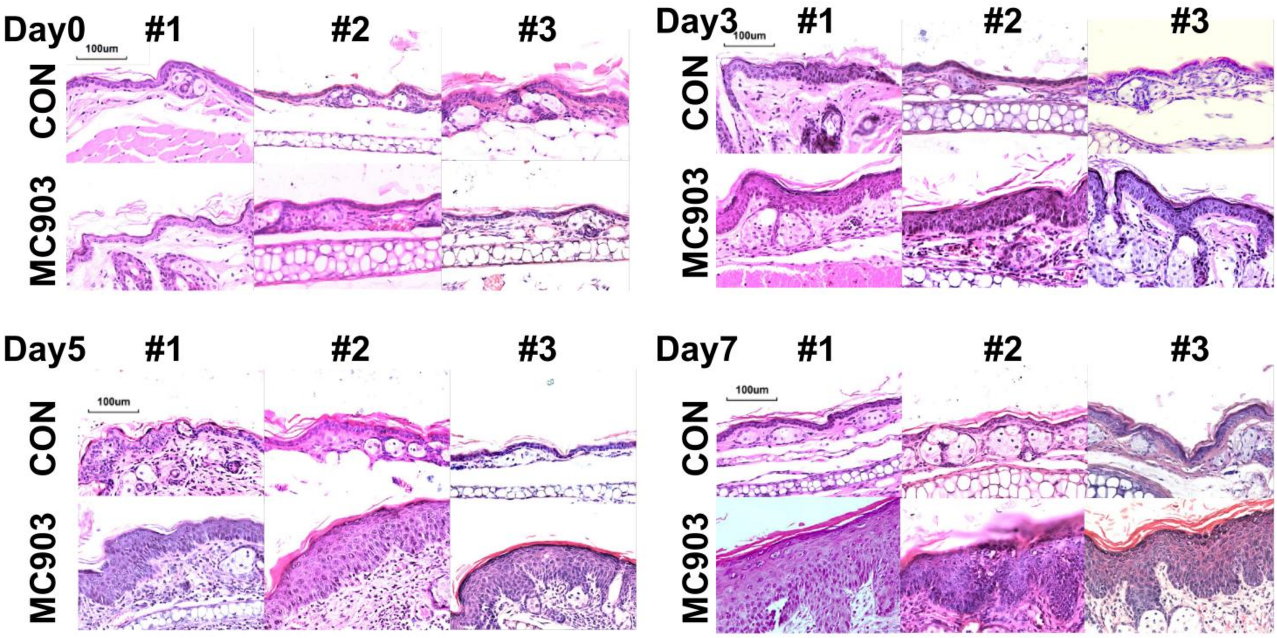
Day0
#1
#2
#3
100um
CON
MC903
Day3
#1
#2
#3
100um
CON
MC903
Day5
#1
#2
#3
100um
CON
MC903
Day7
#1
#2
#3
100um
CON
MC903

Supplementary Figure S4 | Photograph of a large view of the intervention area of the mouse auricle between the CON, MC903, MC903+TAC, and MC903+GLU subgroups in the time points of 7 days **(A)**, 10 days **(B)**, and 15 days **(C)**. Photograph of H&E staining of mouse auricular intervention area tissue between the CON, MC903, MC903+TAC, and MC903+GLU subgroups in the time points of 7 days **(D)**, 10 days **(E)**, and 15 days **(F)**. In this figure, each subgroup contains three independent replicates.

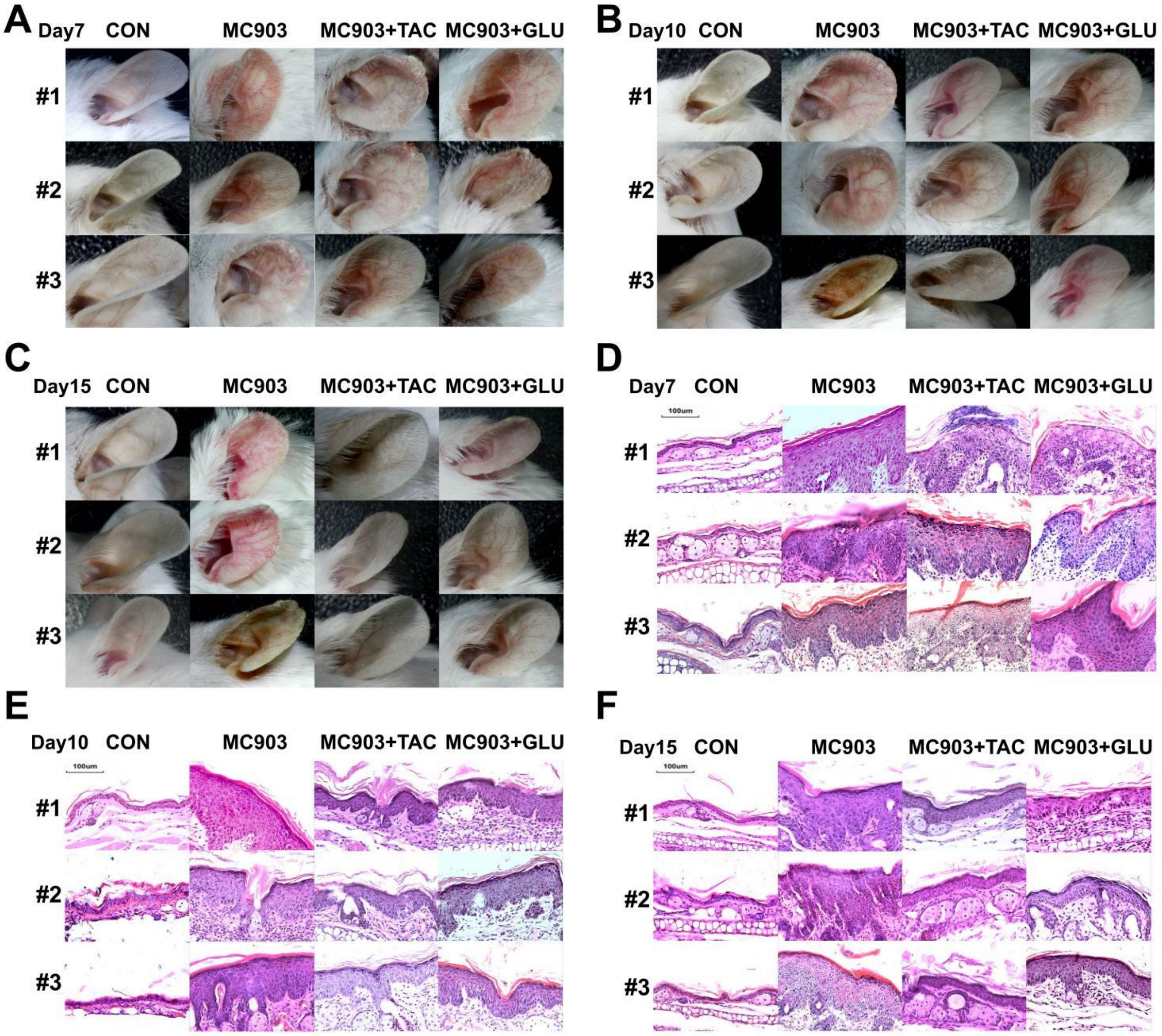

Supplement: Supplementary file 1 [file DataSheet1.pdf]
